# Supplementary material for: Observation of localized magnetic plasmon skyrmions
Source: Nat Commun. 2022 Jan 10;13:8. doi: 10.1038/s41467-021-27710-w (PMC8748431; doi:10.1038/s41467-021-27710-w)
Supplement: Supplementary file 1 — Supplementary Information [file 41467_2021_27710_MOESM1_ESM.pdf]

Supplementary Information:

Observation of Localized Magnetic Plasmon Skyrmions

Zi-Lan Deng[1,2,*], Tan Shi[1], Alex Krasnok[2], Xiangping Li[1,*], Andrea Alù[2,3,*]

[1]Guangdong Provincial Key Laboratory of Optical Fiber Sensing and Communications, Institute of Photonics Technology, Jinan University, Guangzhou 510632, China.
[2]Advanced Science Research Center, City University of New York, New York, NY 10031 USA
[3]Physics Program, Graduate Center, City University of New York, New York, NY 10016, USA

[*]E-mail: zilandeng@jnu.edu.cn, xiangpingli@jnu.edu.cn, aalu@gc.cuny.edu

Supplementary Notes

1. **Model for the space-coiling meta-structure**

A meanderline waveguide model can predict the resonant frequency of the 2D space-coiling cylinder. In this model, the space-coiling structure is equivalent to a metal-insulator-metal perfect electric conductor (PEC) waveguide with a closed-end at one side and an open end at the other side (Fig. S1). The space-coiling cylinder is constructed by a series of semi-circles with gradually increasing radii, and the radius for the ith (i=1,2, …, n) upper semi-circle and i^{th} lower semi-circles are, $r_{i1}=(2i-1)\frac{d}{2}$, $r_{i2}=id$, respectively. The total effective waveguide length of the structure is

$$s=\pi\sum_{i=1}^{n_r}(r_{i1}+r_{i2})=\pi\sum_{i=1}^{n_r}(2id-d/2)=\pi\left(n_r^2 d+\frac{1}{2}n_r d\right), \quad (1)$$

where n_r is the number of turns of the spiral. One side of the waveguide is terminated by a PEC wall. In contrast, the other side is open to the external environment and allows

the coupling between the external field and the waveguide with a finite length. In such a semi-truncated waveguide, the Fabry-Perot (FP) resonance condition gives that,

$$\frac{\lambda_m}{2}+(m\text{-}1)\lambda_m=2s,(m=1,2,...). \tag{2}$$

Therefore, the resonance wavelength of the mth localized spoof plasmon (LSP) mode is,

$$\lambda_m=\frac{4s}{2m\text{-}1},(m=1,2,...), \tag{3}$$

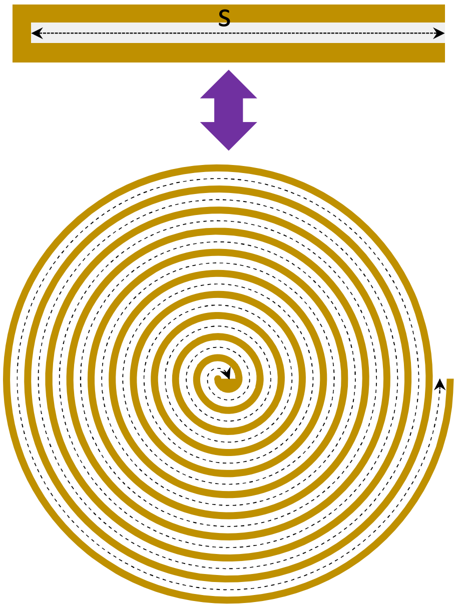

Fig. S1. Equivalent meanderline waveguide model for the space-coiling cylinder. The resonance wavelength depends on the effective waveguide length s.

The resonance condition for the normalized frequency k_0R can be written as,

$$k_0R=\left(m+\frac{1}{2}\right)\pi\frac{R}{s}, \tag{4}$$

substituting $R=n_rd$, and Eq. (1) into Eq. (4),

the resonance frequency for the mth LSP mode yields,

$$k_0R=\left(m+\frac{1}{2}\right)\frac{\pi R}{\pi\left(n_r^2d+\frac{1}{2}n_rd\right)}=\left(m+\frac{1}{2}\right)\frac{\pi n_rd}{\pi\left(n_r^2d+\frac{1}{2}n_rd\right)}=\frac{2m+1}{2n_r+1}, \tag{5}$$

The spacing between adjacent modes, or the free spectral range (FSR) yields,

$$Dk_0R = \frac{2}{2n_r + 1}. \quad (6)$$

Following the above equations, we can roughly predict the resonance positions of each mode and then find the precise resonance peaks by FEM full-wave simulations.

To analyze the modal properties of resonant modes in the near-equidistant spectrum, we can employ the mode expansion approach [1, 2] to show individual angular mode contributions to the scattering cross-section (SCS) spectrum.

When a TM-polarized plane wave is impinging on the space-coiling cylinder, the total field outside the particle (ρ>R) in the cylindrical coordinate can be written as,

$$H_z = H_0 \sum_{n=-\infty}^{\infty} \left[i^n J_n\left(k_0\rho\right) + C_n H_n^{(1)}\left(k_0\rho\right) \right] e^{in\varphi}, \quad (7)$$

where the first term $\sum_{n=-\infty}^{\infty} i^n J_n\left(k_0\rho\right) e^{in\varphi}$ is the incident plane wave, and the second term is the scattering field,

$$H_z^{sca} = H_0 \sum_{n=-\infty}^{\infty} C_n H_n^{(1)}\left(k_0\rho\right) e^{in\varphi}, \quad (\rho>R) \quad (8)$$

where C_n is the mode coefficient for the nth angular mode. n=0 represents the magnetic dipole mode, n=1 represents the electric dipole modes, and n=2 represents the electric quadrupole mode, and so on [3].

While inside the particle (0<=ρ<=R), the field can be written as the superposition of out-going waves and in-going waves as follows,

$$H_z = H_0 \sum_{n=-\infty}^{\infty} B_n^+ H_n^{(1)}\left(k_\rho \rho\right) e^{in\varphi} + B_n^- H_n^{(2)}\left(k_\rho \rho\right) e^{in\varphi}, \quad (0<=\rho<=R), \quad (9)$$

The boundary condition at ρ=0 requires that $B_n^+ = B_n^-$, which leads to a standing wave (along radial direction) within the structure

$$H_z = H_0 \sum_{n=-\infty}^{\infty} A_n J_n\left(k_\rho \rho\right) e^{in\varphi}, \quad (0<=\rho<=R), \tag{10}$$

Multiplying Eq. (8) by $e^{-in'\varphi}$, and integrating it along an arbitrary circle at $\rho = R$ enclosing the particle, it yields,

$$Int = \int_0^{2\pi} H_z^{sca}|_{\rho=R} e^{-in'\varphi} R d\varphi = H_0 \sum_{n=-\infty}^{\infty} \int_0^{2\pi} C_n H_n^{(1)}\left(k_0 R\right) e^{i(n-n')\varphi} R d\varphi . \tag{11}$$

According to the orthogonality between different angular modes, only the n=n' term is nonzero in the right-hand side of the above equation,

$$Int = H_0 C_m H_m^{(1)}\left(k_0 R\right) 2\pi R . \tag{12}$$

As a result, the mode coefficient of the mth angular mode can be expressed as,

$$C_m = \frac{\int_0^{2\pi} H_z^{sca}|_{\rho=R} e^{-im\varphi} R d\varphi}{H_0 H_m^{(1)}\left(k_0 R\right) 2\pi R} . \tag{13}$$

And the SCS owing to the mth mode can be written as,

$$\sigma_m = \frac{4}{k_0} |C_m|^2 . \tag{14}$$

Figure S2 shows a wider frequency range, compared to in Fig. 2(a) in the main text, showcasing the SCS spectra for the first 16 resonant modes, better highlighting the near-equidistant response, as well as the expanded mode contributions calculated with Eq. (12). For all modes, σ_0 is always the dominant contribution term, indicating that all resonant modes are purely magnetic (with angular mode index n=0) with suppressed other electric resonances. Therefore, the field patterns within the space-coiling particle can be described simply by the 0th Bessel function according to Eq. (10),

$$H_z = H_0 A_0 J_0\left(k_\rho \rho\right), \quad (0<=\rho<=R), \tag{15}$$

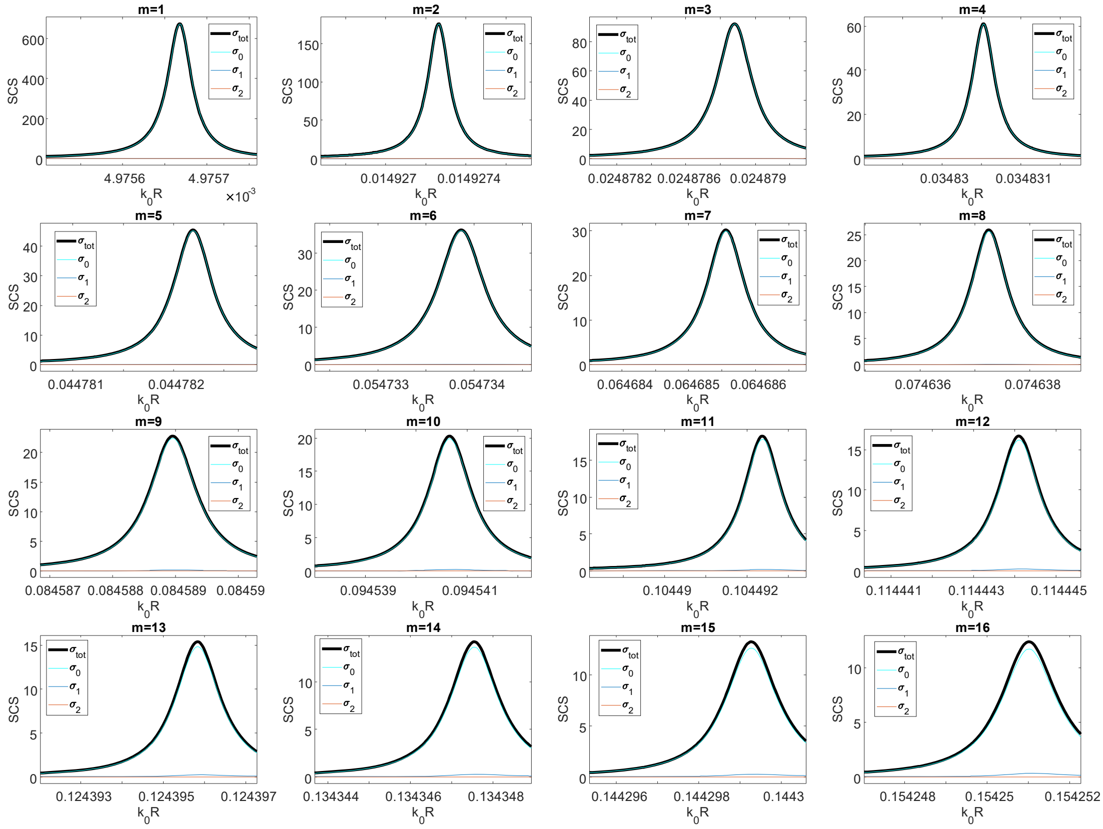

Fig. S2. Angular mode contributions to the SCS of resonant modes in the 2D space-coiling cylinder. The SCS can be divided into contributions of angular modes σ_n with different azimuthal indexes (n=0,1, 2, …). For all the modes in the near-equidistant spectrum, the σ_0 is dominant, manifesting purely magnetic modes.

The scattering properties of the space-coiling cylinder, as shown in Fig. S3a, are equivalent to those of a homogeneous metamaterial. The space-spoiling structure can be considered as a series of dense grooves along the azimuthal direction, resulting in effective parameters [4, 5]:

$$\varepsilon_r = \frac{d}{a},\ \varepsilon_\phi = \infty,\ \varepsilon_z = \infty\,. \tag{16}$$

As EM waves propagate in the grooves along the azimuthal- and z-directions with light velocity, other components of constitution parameters should satisfy,

$$\sqrt{\varepsilon_r \mu_\phi} = \sqrt{\varepsilon_r \mu_z} = 1, \tag{17}$$

therefore

$$\mu_\phi = \frac{a}{d}, \ \mu_z = \frac{a}{d}, \ \text{ and } \mu_r = 1. \tag{18}$$

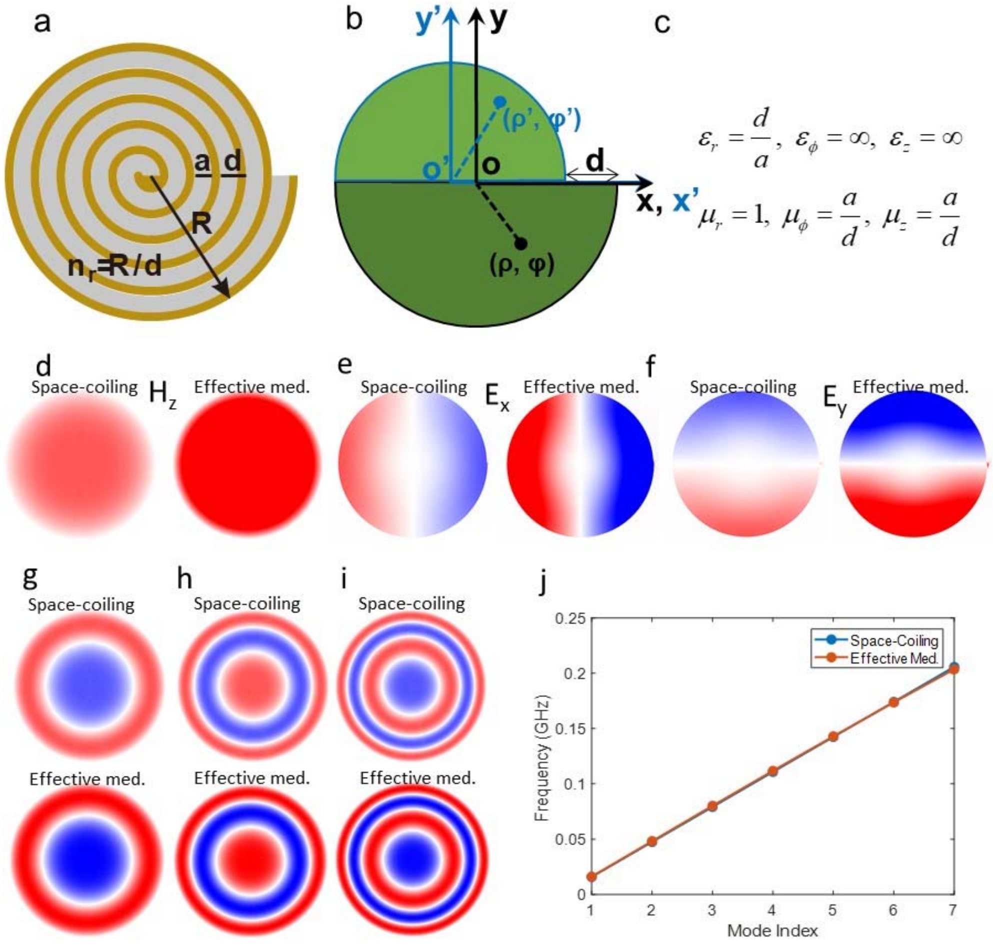

Fig. S3. Effective metamaterial approximation for the space-coiling cylinder. (a) The schematic of the space-coiling cylinder with parameters: gap width a, spiral pitch d, and radius R. (b) The corresponding effective medium of (a), which can be divided into two homogenous semi-circle areas, with slightly different radii R-d and R, respectively, and a small shift between their coordinate centers $oo'=d/2$. The two parts are polarly anisotropic with the same sets of parameters shown in (c), yet defined in different coordinates with respect to their own circle center. (d-f) Comparison of the 0th mode profiles (H_z, E_x, E_y) of the space-coiling cylinder and those of the effective medium. (g-i) Comparison of magnetic (H_z) field profiles of higher modes by the space-coiling cylinder and the effective medium. (j) Resonant frequencies versus mode indexes for space-coiling cylinder and effective medium.

The whole spiral space-coiling cylinder could be divided into two semi-circles with

slightly different radius R and R-d, where d is the spiral pitch (Fig. S3b). Origins of the two semi-circles are slightly shifted with a distance $d/2$ so that they are tangent at one side. The effective parameters for the metamaterial in both semi-circle regions are the same as the ones in Eq. (13) and Eq. (15), except that the coordinates to define those polar anisotropic media have a slight shift $oo'=d/2$. The field patterns of supported resonant modes by the space-coiling cylinder are consistent with those of the effective metamaterial (Fig. S3d-i).

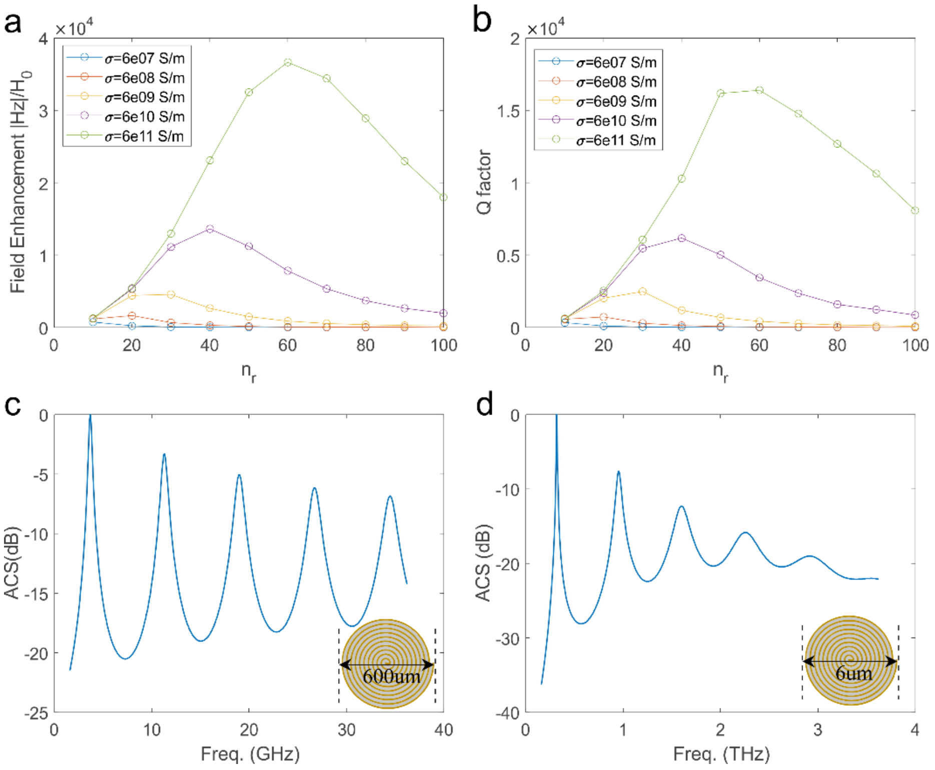

Fig. S4. Influence of dissipation losses on the resonant modes of the near-equidistant multi-resonant spectrum. Field enhancement (at the center of the structure) (a) and quality factors (b) of the 2D space-coiling cylinder with different conductivities σ are plotted as a function of the number of spiral turns n_r. The gap width a and spiral pitch d are set as a/d=2/3, disk radius $R=n_r/d$. (c, d) Resonant spectra of a space-coiling cylinder made of realistic copper material (σ=6e7 S/m) with the diameter (c) 2R=600 microns and (d) 2R=6 microns, respectively. The near-equidistant resonances still preserve with equally-spaced absorption cross-section (ACS) peaks at (c) Gigahertz and (d) Terahertz frequency regimes.

Usually, a metallic structure can be treated as PEC in the low-frequency range, including Terahertz and microwave. However, in our present study, the resonances of dense space-coiling structures are extremely strong. Thus, the field enhancement and quality factors are highly sensitive to dissipation losses. When the metallic dissipation loss is considered, the field enhancement and quality factor of the resonant modes no longer follow the ideally linear increasement with respect to the number of turns n_r as shown in Fig. 2e in the main text. Instead, they first increase with n_r, and then saturate at a finite n_r, and after that, they begin to decline (Figs. S4a, b). The larger the metal conductivity (the smaller the dissipation loss), the bigger n_r to reach the peak field enhancement and quality factor. Therefore, we should optimize n_r to balance between the field confinement and the field enhancement in the practical situation. In our experiment, we choose n_r=20. The field confinement already reaches an extremely small region down to $\lambda^3/10^6$. At the same time, the field enhancements are strong enough to be measured as clearly sharp peaks, as demonstrated in Fig. 3c in the main text. Figures S4c and d show the scaling of the space-coiling cylinder made of realistic copper material (σ=6e7 S/m) to 600-micron and 6-micron diameters, which demonstrate the comb-like resonances at an extremely deep-subwavelength scale near 10GHz and 1THz respectively, promising for extremely miniaturized GHz and THz devices.

2. Finite height space-coiling meta-structures

For the 2D space-coiling cylinder with an infinite height, the localized magnetic field

exhibits scalar profiles with only the z-component, and the localized electric field is restricted to the in-plane x-, and y-components. When it evolves into a finite height structure, an evanescent wave with the full vectorial characteristic of both magnetic and electric fields emerges at two end-facets of the cylinder (Fig. S5). In the meta-structure area with an extreme anisotropic effective medium, the supported mode is highly squeezed ($k_\rho >> k_0$). In the air area with normal constitute parameters, the dispersion relation of light is $k_\rho^2 + k_z^2 = k_0^2$, therefore $k_z = \sqrt{k_0^2 - k_\rho^2}$ is a complex number with a huge imaginary part, indicating rapid field decaying in the z-direction. Assuming the decaying constant along the z-direction is $\beta_z = -ik_z = \sqrt{k_\rho^2 - k_0^2}$, we can write the field profiles near the interface between the meta-structure and air as,

$$H_z = H_0 A_0 J_0\left(k_\rho \rho\right) e^{-\beta_z z}, \tag{19a}$$

$$H_x = H_0 A_0 \frac{-\beta_z}{k_\rho} J_0^{'}\left(k_\rho \rho\right) e^{-\beta_z z} \frac{x}{\rho}, \tag{19b}$$

$$H_y = H_0 A_0 \frac{-\beta_z}{k_\rho} J_0^{'}\left(k_\rho \rho\right) e^{-\beta_z z} \frac{y}{\rho}. \tag{19c}$$

The corresponding unit vector field $h_i = \dfrac{H_i}{\sqrt{H_x^2 + H_y^2 + H_z^2}}, \left(i = x, y, z\right)$ can be written as,

$$h_z = J_0\left(k_\rho \rho\right) / \sqrt{J_0^2\left(k_\rho \rho\right) + \frac{\beta_z^2}{k_\rho^2} J_0^{'2}\left(k_\rho \rho\right)}, \tag{20a}$$

$$h_x = \frac{\beta_z^2}{k_\rho^2} J_0^{'2}\left(k_\rho \rho\right) \frac{x}{\rho} / \sqrt{J_0^2\left(k_\rho \rho\right) + \frac{\beta_z^2}{k_\rho^2} J_0^{'2}\left(k_\rho \rho\right)}, \tag{20b}$$

$$h_y = \frac{\beta_z^2}{k_\rho^2} J_0^{'2}\left(k_\rho \rho\right) \frac{y}{\rho} / \sqrt{J_0^2\left(k_\rho \rho\right) + \frac{\beta_z^2}{k_\rho^2} J_0^{'2}\left(k_\rho \rho\right)}. \tag{20c}$$

Set $\varphi = \tan^{-1}\left(\frac{y}{x}\right)$, $\Theta(\rho) = \tan^{-1}\left(\frac{\beta_z}{k_\rho}\frac{J_0^{'}(k_\rho\rho)}{J_0(k_\rho\rho)}\right)$, then,

$$\begin{pmatrix} h_x \\ h_y \\ h_z \end{pmatrix} = \begin{pmatrix} \cos\varphi\sin(\Theta(\rho)) \\ \sin\varphi\sin(\Theta(\rho)) \\ \cos(\Theta(\rho)) \end{pmatrix}, \tag{21}$$

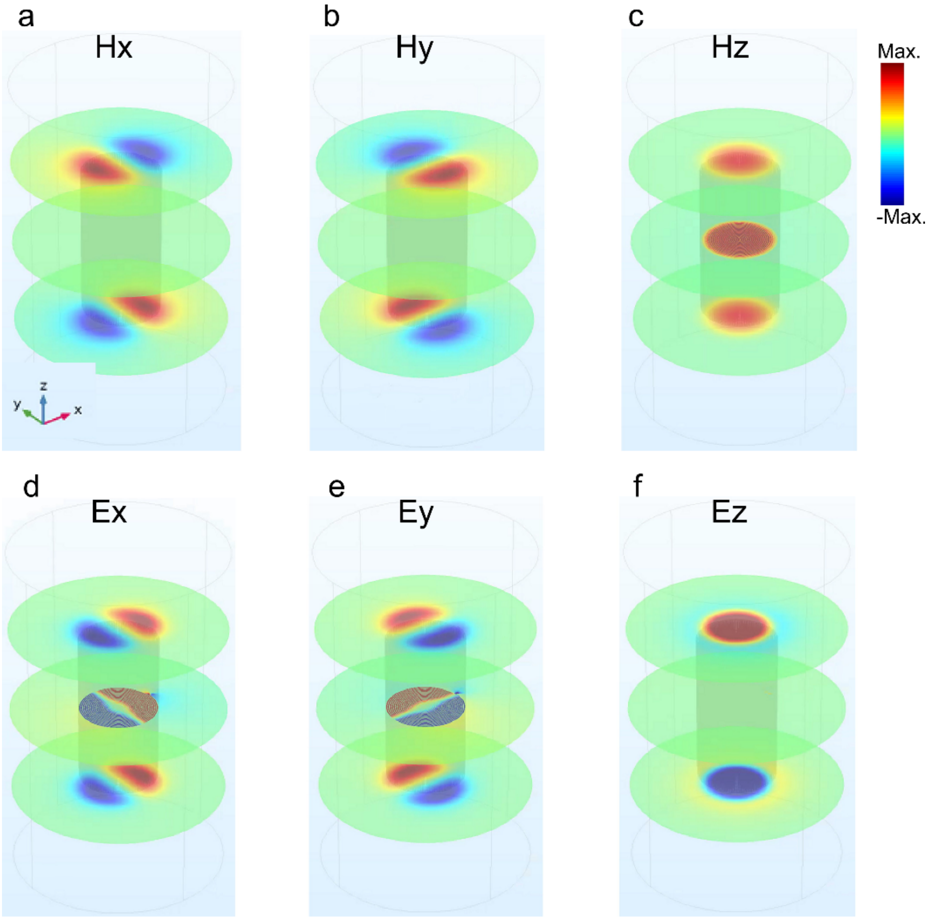

Fig. S5. Field patterns of a finite height space-coiling cylinder. In the central area of the cylinder, only H_z, E_x, E_y components are nonzero, which is consistent with the 2D case. At the end facets of the cylinder, 3D vectorial configurations are present. The skyrmion number of the unit magnetic field vectorial configuration is calculated as 1, which indicates an elementary skyrmion field configuration. The gap width a and spiral pitch d are set as a/d=2/3, the number of rings is n_r=20, and the disk radius R=n_r/d.

which is the same form as Eq. (2) in the main text. Consequently, the skyrmion number of the resonant modes can be analytically calculated, dependent on only the value of $\Theta(\rho)$ in the central point and that in the periphery. For the fundamental mode,

$\Theta(\rho=0)$=0 at the central point, and $\Theta(\rho=R)$=π at the periphery, and therefore the skyrmion number is 1, which can be further confirmed by numerically calculating the skyrmion number of the vectorial magnetic field distributions in Fig. S5a-c. The LSP mode always exists when gradually reducing the height, although the resonance peaks blue-shift (Fig. S6a). The fields are tightly confined near the structure even if the height vanishes to become a metasurface structure (Fig. S6b).

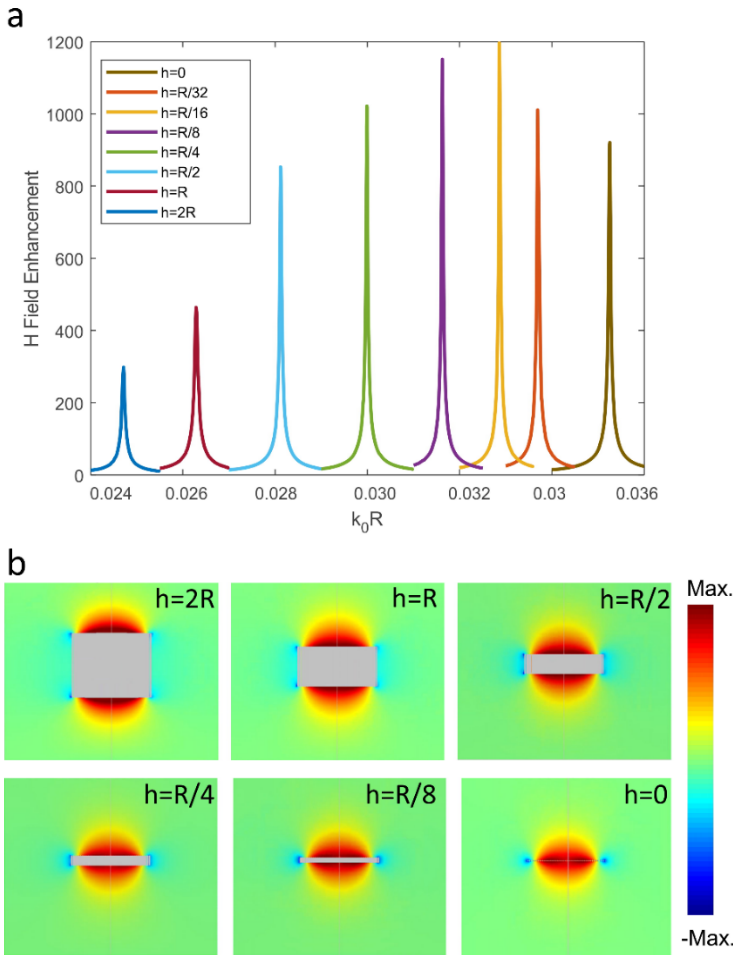

Fig. S6. Transition of the resonant modes by decreasing the height of the space-coiling cylinder. (a) The resonant spectra of the fundamental modes for different cylindric heights. As the height decreases, the resonant peak shifts towards higher frequencies. Even when the height vanishes (h=0), the localized resonance mode still exists. (b) The corresponding magnetic field enhancement profiles ($|H_z|/H_0$, xz-plane) of those modes for different cylinder heights. The gap width a and spiral pitch d are set as a/d=2/3, disk radius R=n_r/d.

3. Robust excitation of LSP skyrmions with near-equidistant multi-resonant response

In previously demonstrated optical analogs of skyrmions based on the interference of propagating surface plasmon polaritons[6, 7], the skyrmion field configuration is constructed by externally manipulating the field structure, which is highly dependent on the excitation conditions. In ref. [6], a bulky tight focusing optical setup was employed to illuminate structured light on a metallic film. The optical spin configuration of a skyrmion-type distribution was formed in the focusing area. In ref. [7], the interference of multiple illumination propagating surface waves was employed. Multiple sets of metallic slit arrays were etched in a metallic film to excite the propagating surface plasmon waves with proper propagation directions and initial phases. Consequently, skyrmion lattice was generated in the central area of the smooth metal film. Both wavelengths and incident angle highly restrict the excitation condition.

In contrast, our approach generates inherent LSP skyrmions. The skyrmions are inherent resonant modes of the space-coiling metasurface, which are completely independent of specified excitation configurations. The excitation of those inherent skyrmion modes can be achieved by various EM sources such as point sources (Fig. S7a) or plane wave sources (Fig. S7b). The excitation conditions are largely relaxed. The robust excitations of these inherent skyrmions are sensitive to neither the source position nor the incident direction, albeit the excitation efficiency is varied.

These skyrmions can be flexibly designed on-demand by structure parameters, such as the size, shape and frequency and the construction of arbitrary forms of arrays,

forming a periodic metasurface with tailored far-field polarization or chirality manunipuations[8, 9]. Such design flexibility is crucial to promote the skyrmions for practical applications such as ultra-compact frequency combs, robust laser cavities, random-access memories, wireless power transfers and microwave photonics.

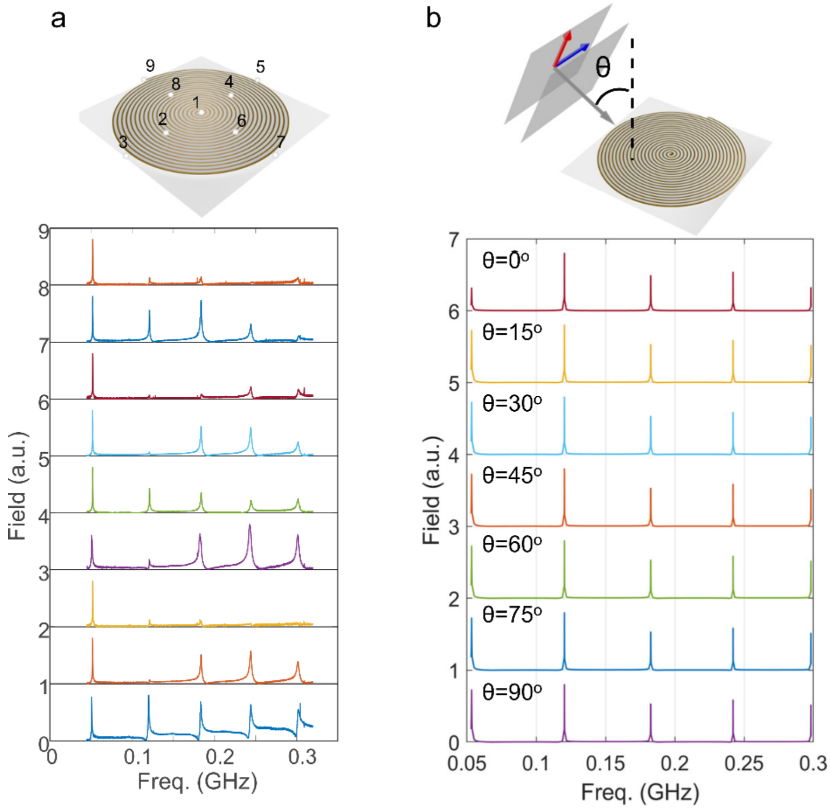

Figure S7. Robust excitation of the LSP skyrmions at various conditions (a) Experimental measurement of the near-equidistant spectra by point source excitations of the space-coiling metasurface at different positions. (b) Calculated near-equidistant spectra by plane wave illuminations on the space-coiling metasurface at different incident angles. When the excitation condition changes, all the skyrmion modes can be reliably generated. And the resonant peak positions in the near-equidistant spectrum remain the same, although the mode strengths may differ due to different coupling efficiencies between the excitation source and a specified inherent mode of the metasurface.

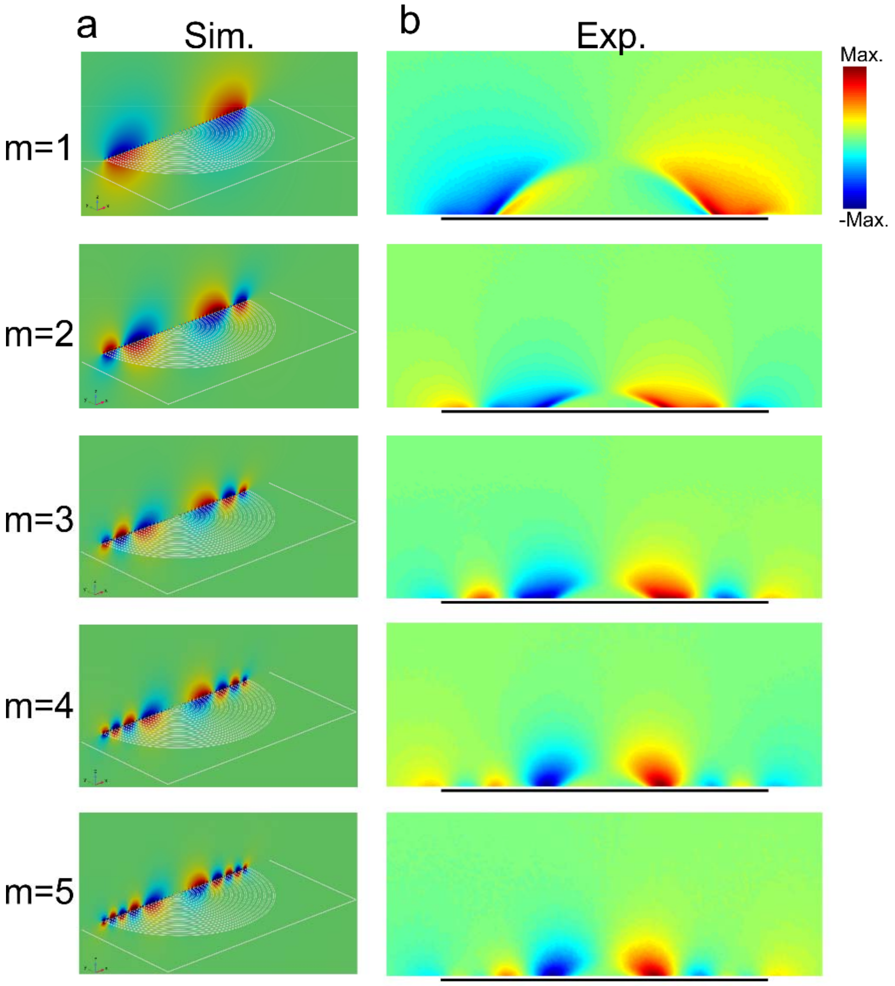

Figure S8. In-plane field (H_x) distribution at a vertical cross-section of the space-coiling metasurface. Simulated (a) and measured (b) H_x field patterns for modes m=1~5 in the xz-plane are shown at their resonant frequencies. Fields are well confined near the interface.

4. Comparison of the topological features of magnetic and electric field vectorial configurations of the resonant modes

While the magnetic field profile exhibits full skyrmion vectorial configurations, the electric field profile exhibits a combination of an elementary skyrmion formed by the central mode lope (electric field tip evolving over a π-twist), and an additional π/2-twist, in the periphery (Fig. S9a). The corresponding skyrmion density is shown in Fig. S9b. The cosine function of the electric field orientation angle indicates that the whole

vectorial configuration experiences skyrmion number 1 in the central and -1/2 in the peripheral mode lobes, resulting in a total skyrmion number of 1/2 [10, 11].

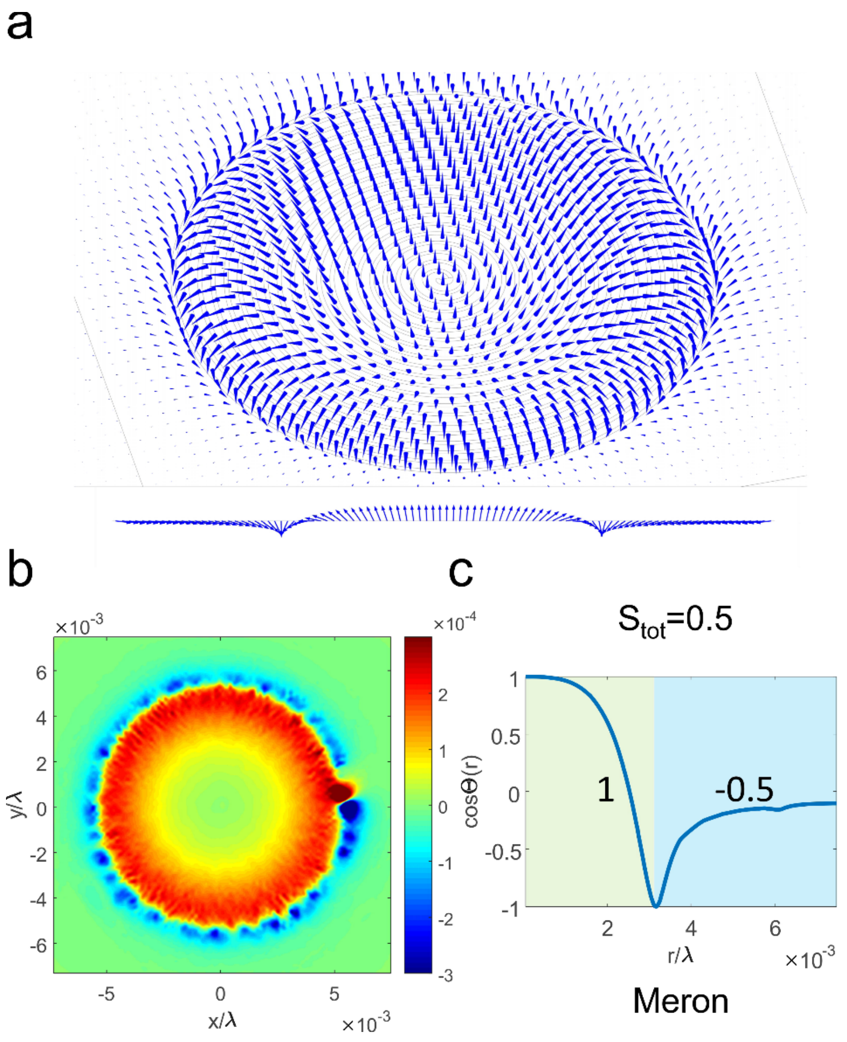

Figure S9. Vectorial electric field profile of the resonant mode in the space-coiling metasurface. (a) Vectorial configuration of the electric field (up) and the unit vector configuration in the radial direction (down). The field points up in the center and points in the horizontal direction in the perimeter (b) skyrmion density of the electric field vector configuration. (c) The Cosine of the electric field orientation angle along the radial direction, showing a total skyrmion number 1/2.

It seems strange that both electric and magnetic fields point to the same radial direction in the central area of the skyrmion mode. This is because the resonant mode is in principle a standing wave formed by outward and inward surface waves along the radial direction. As a result, there is a $\pi/2$ phase between the electric field and magnetic field

(Fig. S10). The dynamic evolutions of the magnetic and electric field vectorial configurations are demonstrated in Movie S1. The maxima of the two fields alternatively appear in time sequence. Nevertheless, the local tip in the vectorial configurations always points in the same or opposite direction without any rotation, which indicates that they are stable and well-defined skyrmion-related vectorial configurations all the time. The stability of the multiple-π-twist skyrmion modes is also demonstrated in Movie S2.

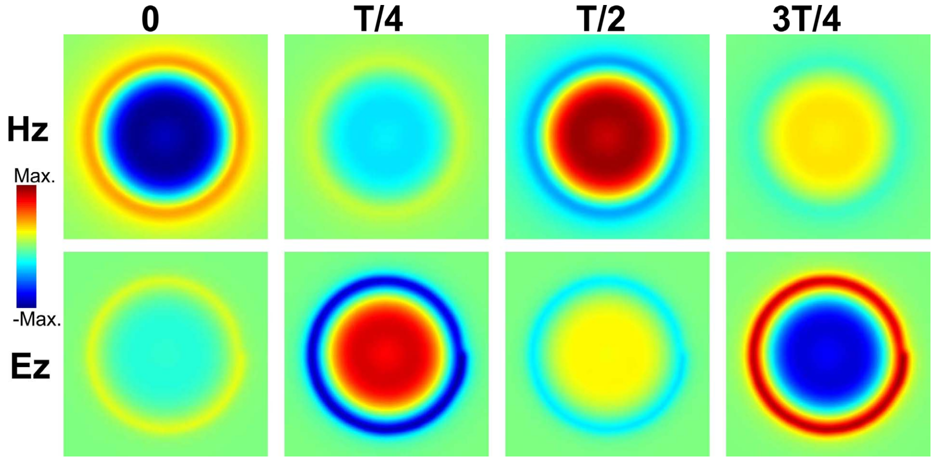

Figure S10. π/2 phase retardation between the electric field and magnetic field, due to the standing wave nature of the resonant surface waves along the radial direction. The figures depict field profiles in different phase delays within a time cycle, where T is the period of the time sequence.

5. Skyrmion density and number of different shaped LSP skyrmions

To validate the skyrmion density and skyrmion number calculation, we compared the unit magnetic field profiles and the skyrmion density profiles between simulation and experiment, as shown in Fig. S11. The measured unit vector components $\left(h_x, h_y, h_z\right) = \left(H_x, H_y, H_z\right) / |\mathbf{H}|$ have been post-processed with a low-pass frequency filter to remove high-frequency noises. Compared with the simulation results, the size of the central null area of the h_x and h_y patterns are larger than expected in simulations

(Fig. S11 e, f), which is due to the strong coupling between the space-coiling meta-structure and the loop antenna. The extracted skyrmion density is shown in Fig. S11 h, and it is a ring-like pattern, consistent with the simulation pattern in Fig. S11 d. The integration of the skyrmion density yields a skyrmion number equal to 0.9905, consistent with the simulation skyrmion number of 0.9930. Therefore, the consistency between measured and simulated vectorial magnetic field patterns, and the skyrmion density retrieved from the experimental data, fully confirm the topological traits predicted in our model.

When the space-coiling metasurface is deformed from the ideal circular shape to any other shapes, such as polygons, ellipse, and even asymmetric heart shapes (Fig. S12a), the resonant modes maintain the hedgehog-like vectorial field configurations (Fig. S12b), and the overall field profiles adapt to their corresponding geometric shapes. The skyrmion densities derived from those vectorial field configurations exhibit distinct distributions (Fig. S12c) with their maxima distributed at sharp corners with large curvatures. However, the numerical integration of those skyrmion densities gives similar quantities close to 1, which represents the topological invariant (skyrmion number) within a reasonable tolerance range due to numerical error. Note that, the demonstrated skyrmion topology manifests hedgehog-like vectorial field configurations over a 2D plane, and such topology is observed overall xy-plane cross-sections of the surface mode profile, independent of z. Its topological robustness is rooted into the skyrmion features, fundamentally different from the **E**-field distribution over a PEC boundary. For instance, a PEC sphere with radially distributed **E**-fields

along its 3D surface does not support a skyrmion topology, and the PEC boundary only restricts a hedgehog-like **E**-field profile on the boundary, not for all fields across the mode volume. The corresponding topological features are trivial, and the response of such structure is not robust to shape deformations.

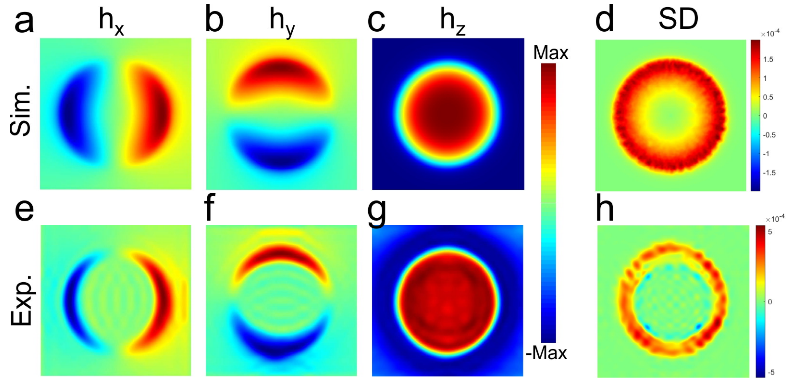

Figure S11. Comparison between the simulated (a-d) and measured (e-h) unit magnetic field components (h_x, h_y, h_z)=(H_x, H_y, H_z)/|H| and skyrmion density (SD) of the elementary skyrmion mode. The extracted skyrmion numbers are 0.9930 and 0.9905 from simulation and experiment, respectively.

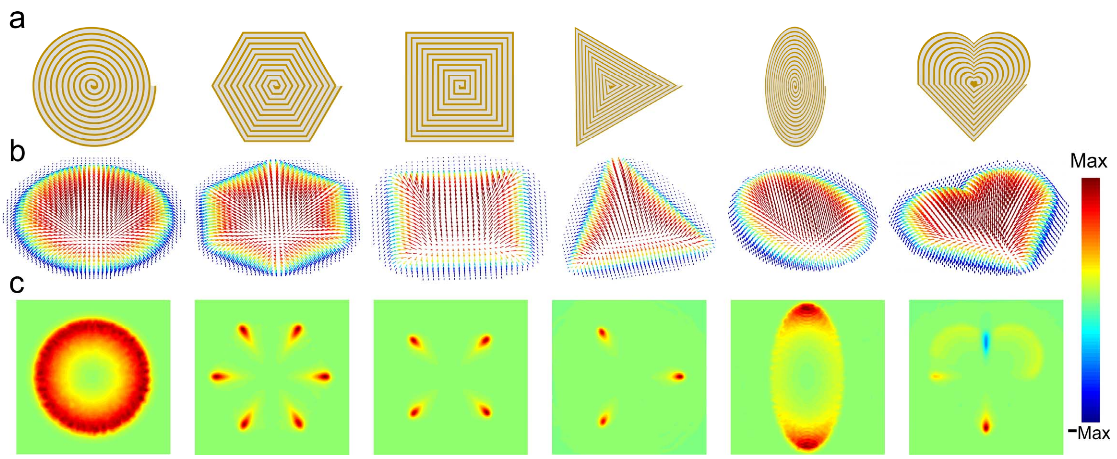

Figure S12. Field characteristic of the fundamental resonant mode when the space-foiling metasurface is continuously deformed to a variety of shapes. (a) Schematics of the space-coiling geometries, with duty circle a/d=2/3, and the number of rings n_r=20. (b) simulated magnetic vectorial configurations of the skyrmion mode, and (c) skyrmion density distributions for circle-shaped, hexagon-shaped, square-shaped, triangle-shaped, ellipse-shaped, and heart-shaped geometries (from left to right). Although the distributions of skyrmion densities are different and tend to accumulate in sharp corners, the calculated skyrmion numbers by integrating the skyrmion densities yield to S=0.9930, S=0.9929, S=0.9940, S=0.9925, S=0.9971 and S=0.9939, respectively, revealing the topological invariant.

Figure S13 shows the skyrmion density distributions of multiple-order modes for a variety of geometry shapes. The skyrmion densities are always concentrated at places where the largest curvature takes place. For higher-order modes, the skyrmion densities exhibit alternative positive and negative magnitudes from the center to the periphery, yielding the skyrmion number 1 for odd modes and 0 for even modes for different shaped structures (Figure S14a). Also, the resonant frequencies and overall near-equidistant spectrum remain stable when the cavity shapes are deformed, as shown in Figure S14b.

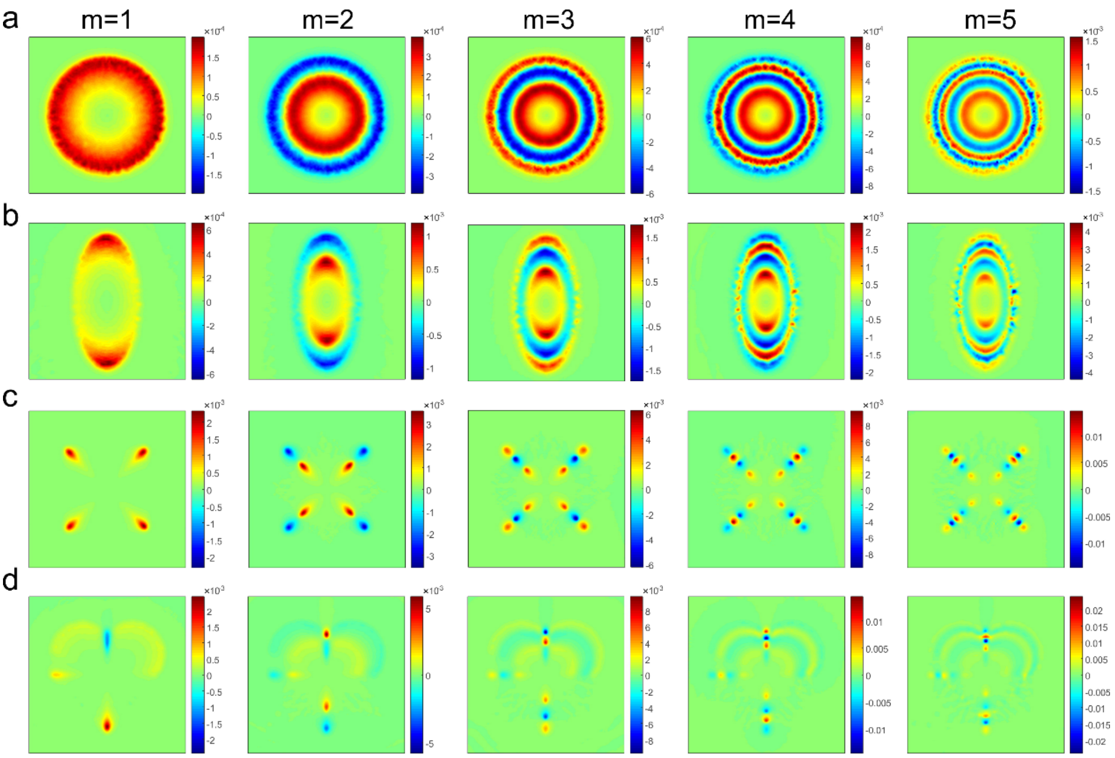

Figure S13. Magnetic skyrmion density distribution of multiple-order resonant modes for various shaped space-coiling metasurfaces with duty circle a/d=2/3, and the number of rings n_r=20, ranging from (a) circle to (b) ellipse, (c) square, and (d) heart shape.

To experimentally demonstrate the robustness of those skyrmion topologies, we fabricated different kinds of space-coiling structures: an ellipse, a square and an

asymmetric heart-shape, and measured the field profiles for the three magnetic field components of their resonant modes, as shown in Figs. S15-S17. The in-plane field components point towards directions that are perpendicular to the polygon edges. Therefore, H_x and H_y reveal nodal-line profiles along their perpendicular axis (y-axis and x-axis, respectively). All the out-of-plane field profiles reveal closed rings following their geometric shapes. Both in-plane and out-of-plane mode profiles have the same number of mode lobes along the radial direction given by the mode index, which are consistent with the simulation results as shown in Fig. 5 of the main text.

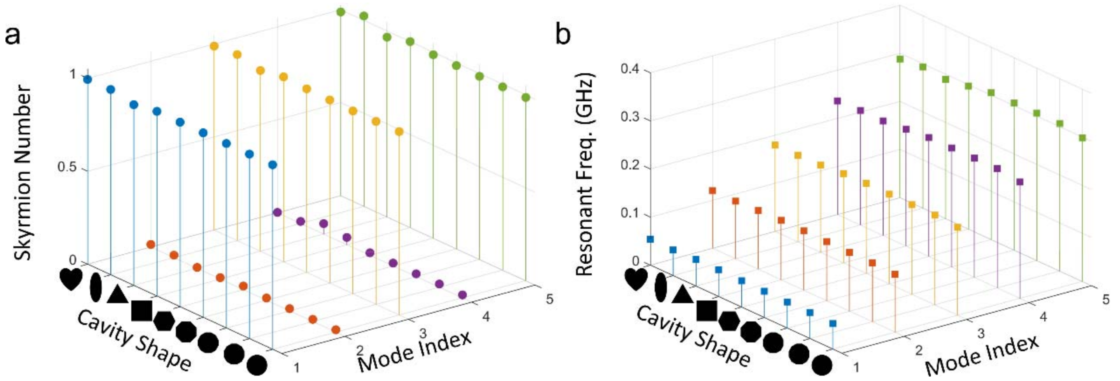

Figure S14. Skyrmion numbers of magnetic field (a) and spectral response (b) of the resonant modes for various shaped space-coiling metasurfaces from the heart shape, ellipse, triangle, square, hexagon, octagon, hexadecagon, icosagon to circle. The skyrmion numbers are always close to 1 (0) for odd (even) modes, irrespective of the geometric shape of the metasurfaces. The resonance frequencies of all modes in the near-equidistant spectra also sustain stability against geometry deformations.

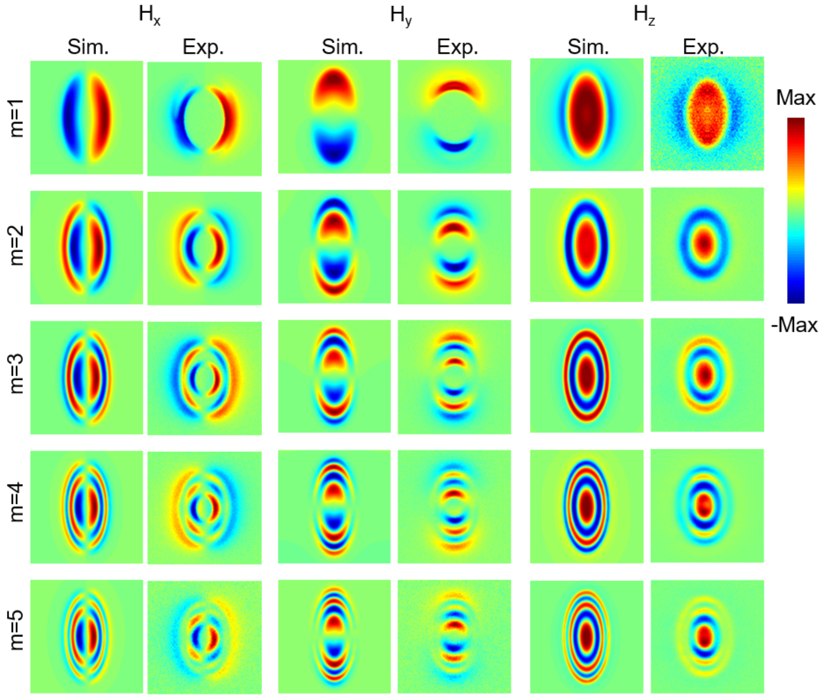

Figure S15. Comparison between simulated and measured all vectorial components (H_x, H_y, H_z) of magnetic field patterns in the ellipse-shaped LSP skyrmions.

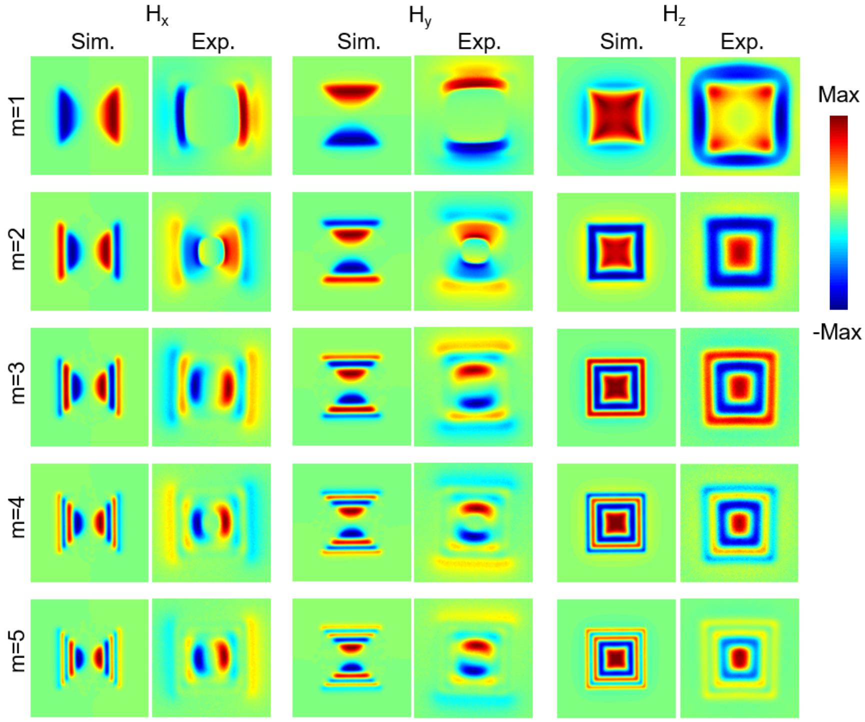

Figure S16. Comparison between simulated and measured all vectorial components (H_x, H_y, H_z) of magnetic field patterns in the square-shaped LSP skyrmions.

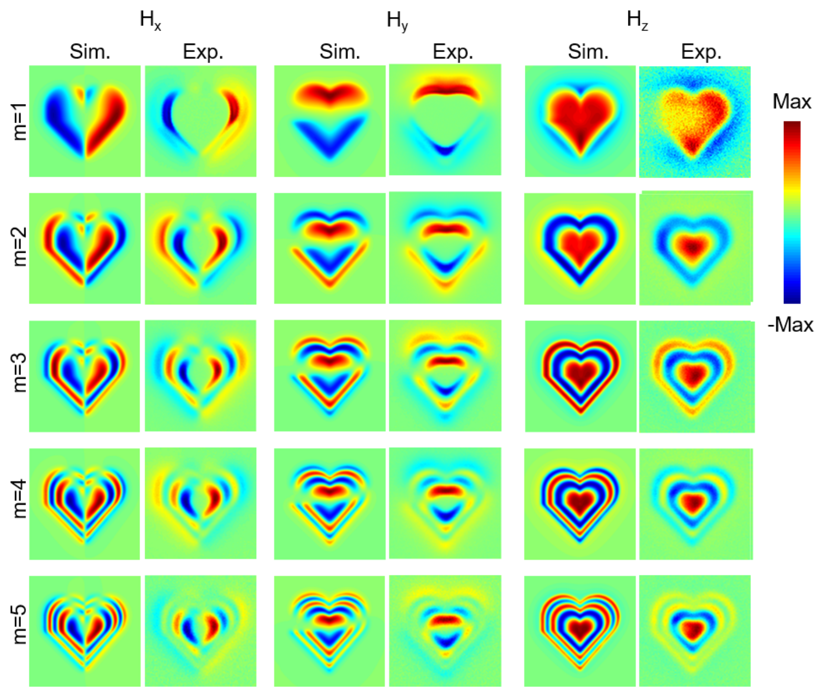

Figure S17. Comparison between simulated and measured all vectorial components (H_x, H_y, H_z) of magnetic field patterns in the asymmetric heart-shaped LSP skyrmions.

6. Overall near-equidistant multi-resonant spectrum of LSP skyrmions

The equivalence to meanderline waveguides explains quite elegantly the evenly-spaced multi-resonance features of our skyrmion structure. In the main text, we experimentally show the first several representative modes with nearly-equidistant resonance frequencies. Because higher-order modes are more sensitive to absorption losses in the metal, the resonances become weaker, the peak values of the near-equidistant response become smaller and finally vanish at higher frequencies, as demonstrated in Fig. S18. The resonant frequencies show a near-linear dependence with the mode index. However, due to other factors such as scattering loss, the dispersion of lossy metal, and the dielectric substrate, the frequency spacing between resonant modes observed in the experiments is not as strictly equi-spaced as that in a frequency comb, the dependence

of resonant frequency on the mode index is approximatively a linear relation.

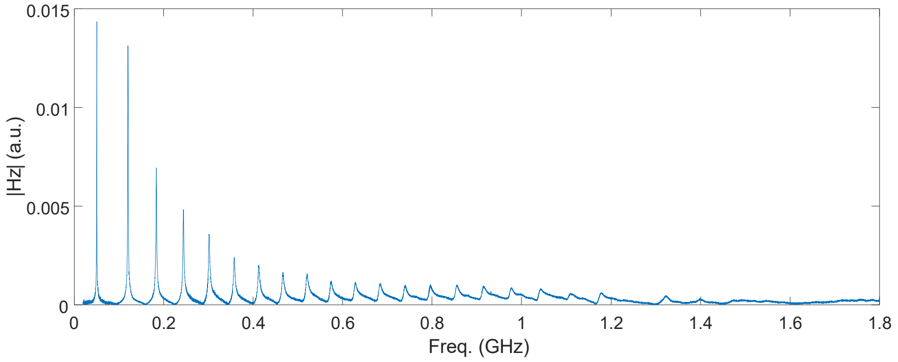

Figure S18. Measured overall near-equidistant spectrum of the LSP skyrmion.

7. Effect of non-continuous defects perturbing the meanderline waveguide

In the previous sections, we have discussed the robustness of the skyrmion topology in our space-coiling meta-structure against continuous shape deformations. In this section, we test the skyrmion topology robustness to defects that abruptly modify the continuity of the space-coiling meta-structure, which significantly affects the meanderline waveguide geometry. For gap perturbations that break the continuity of the meanderline (Supplementary Figs. S19 a, b), no matter how small the void defect or how long of a defect running through multiple channels of the waveguide, the field profiles only expand over a little larger volume, while the continuity of the field profile is not affected. The skyrmion densities are therefore still ring-like distributed, only with different radii and ring widths. The resulting skyrmion numbers are therefore unaffected, indicating that the skyrmion topology is robust against gap perturbations of the space-coiling meta-structure.

On the other hand, interruptions of the meanderline by connecting multiple arms with shorts will abruptly change the field configuration (Supplementary Figs. S19 c, d). Such

defects split the space-coiling waveguide into multiple segments, blocking the continuous propagation of the effective meanderline waveguide mode. The derived skyrmion density distributions are hence also dramatically affected, with opposite values arising near the defect. Although the skyrmion number for small defects of this type is still close to 1, it does not maintain the same value when the length of the defect increases.

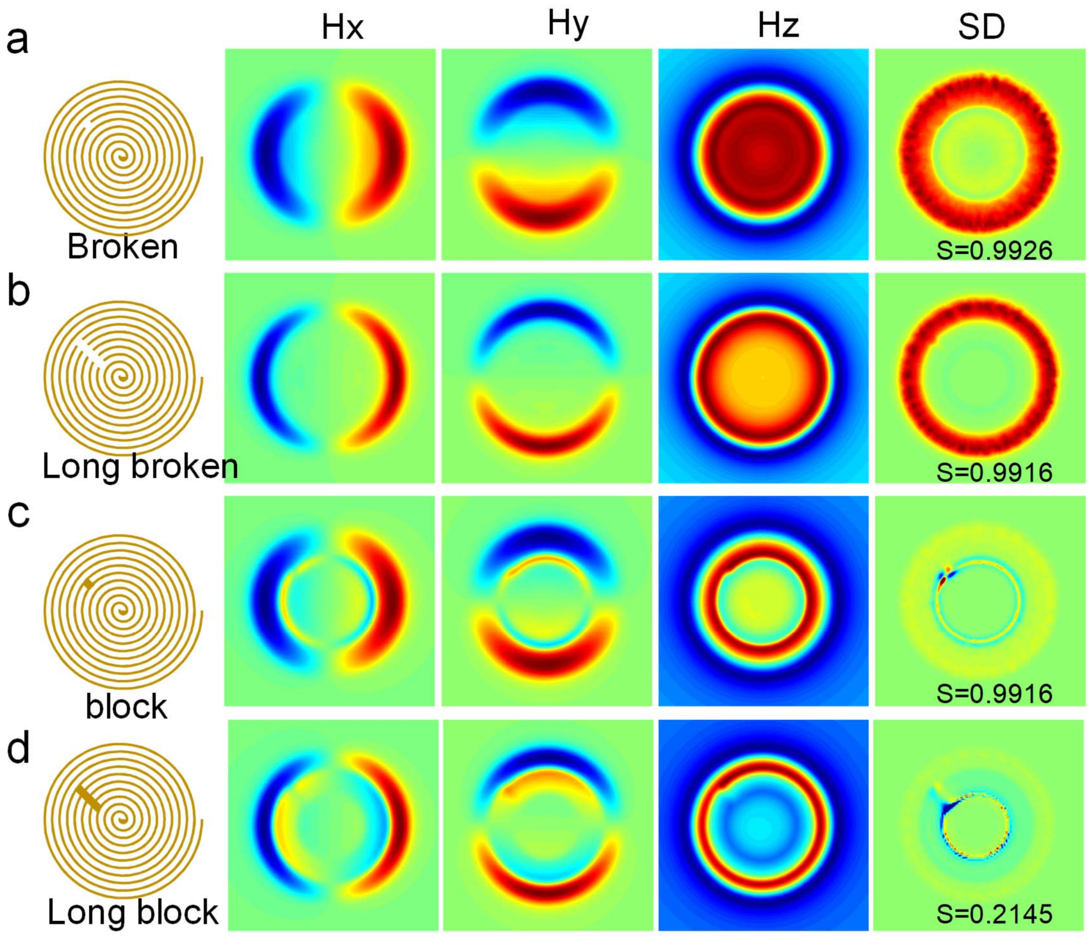

Figure S19. Field configurations, skyrmion densities and skyrmion numbers of the fundamental resonant mode when the space-coiling meta-structure is subjected to different types of defects: (a) a gap perturbation by a broken path running through a single waveguide channel; (b) a long gap perturbation by a broken path running through multiple channels; (c) an interruption by a block defect running through a single waveguide channel; (d) an interruption by a long block defect running through multiple channels.

Supplementary Movies 1. Dynamic evolutions of the magnetic field (red) and electric field vectorial configurations (blue) of the elementary skyrmion mode.

Supplementary Movies 2. Dynamic evolution of the magnetic field unit vectorial configurations of modes 1 to 5 along the radial direction of the LSP skyrmions.

References

1. Grahn, P., Shevchenko, A. & Kaivola, M. Electromagnetic multipole theory for optical nanomaterials. *New J. Phys.* **14**, 093033 (2012).
2. Liao, Z.*, et al.* Homogenous Metamaterial Description of Localized Spoof Plasmons in Spiral Geometries. *ACS Photon.* **3**, 1768-1775 (2016).
3. Liu, W. & Miroshnichenko, A. E. Beam Steering with Dielectric Metalattices. *ACS Photon.* **5**, 1733-1741 (2018).
4. Pendry, J. B., Martín-Moreno, L. & Garcia-Vidal, F. J. Mimicking Surface Plasmons with Structured Surfaces. *Science* **305**, 847-848 (2004).
5. Garcia-Vidal, F. J., Martín-Moreno, L. & Pendry, J. B. Surfaces with holes in them: new plasmonic metamaterials. *J. Opt. A: Pure Appl. Opt.* **7**, S97 (2005).
6. Du, L., Yang, A., Zayats, A. V. & Yuan, X. Deep-subwavelength features of photonic skyrmions in a confined electromagnetic field with orbital angular momentum. *Nat. Phys.* **15**, 650-654 (2019).
7. Tsesses, S.*, et al.* Optical skyrmion lattice in evanescent electromagnetic fields. *Science* **361**, 993-996 (2018).
8. Krasavin, A. V.*, et al.* Polarization conversion and “focusing” of light propagating through a small chiral hole in a metallic screen. *Appl. Phys. Lett.* **86**, 201105 (2005).
9. Reichelt, M.*, et al.* Broken enantiomeric symmetry for electromagnetic waves interacting with planar chiral nanostructures. *Applied Physics B* **84**, 97-101 (2006).
10. Yu, X. Z.*, et al.* Transformation between meron and skyrmion topological spin textures in a chiral magnet. *Nature* **564**, 95-98 (2018).
11. Guo, C., Xiao, M., Guo, Y., Yuan, L. & Fan, S. Meron Spin Textures in Momentum Space. *Phys. Rev. Lett.* **124**, 106103 (2020).
